# Supplementary material for: Cellular Membrane Localization of Innate Immune Checkpoint Molecule CD47 Is Regulated by Radixin in Human Pancreatic Ductal Adenocarcinoma Cells
Source: Biomedicines. 2023 Apr 7;11(4):1117. doi: 10.3390/biomedicines11041117 (PMC10136002; doi:10.3390/biomedicines11041117)
Supplement: Supplementary file 1 [file biomedicines-11-01117-s001.zip › biomedicines-2304493-supplementary.pdf]

biomedicines

Supplementary Materials

Cellular Membrane Localization of Innate Immune Checkpoint Molecule CD47 is Regulated by Radixin in Human Pancreatic Ductal Adenocarcinoma Cells

Takuro Kobori [1], Yui Ito [1], Yuka Sawada [1], Yoko Urashima [1], Takuya Ito [2] and Tokio Obata [1,*]

[1] Laboratory of Clinical Pharmaceutics, Faculty of Pharmacy, Osaka Ohtani University, Tondabayashi, Osaka 584-8540, Japan

[2] Laboratory of Natural Medicines, Faculty of Pharmacy, Osaka Ohtani University, Tondabayashi, Osaka 584-8540, Japan

* Correspondence: obatatoki@osaka-ohtani.ac.jp; Tel.: +81-721-24-9371

Original immunoblot images

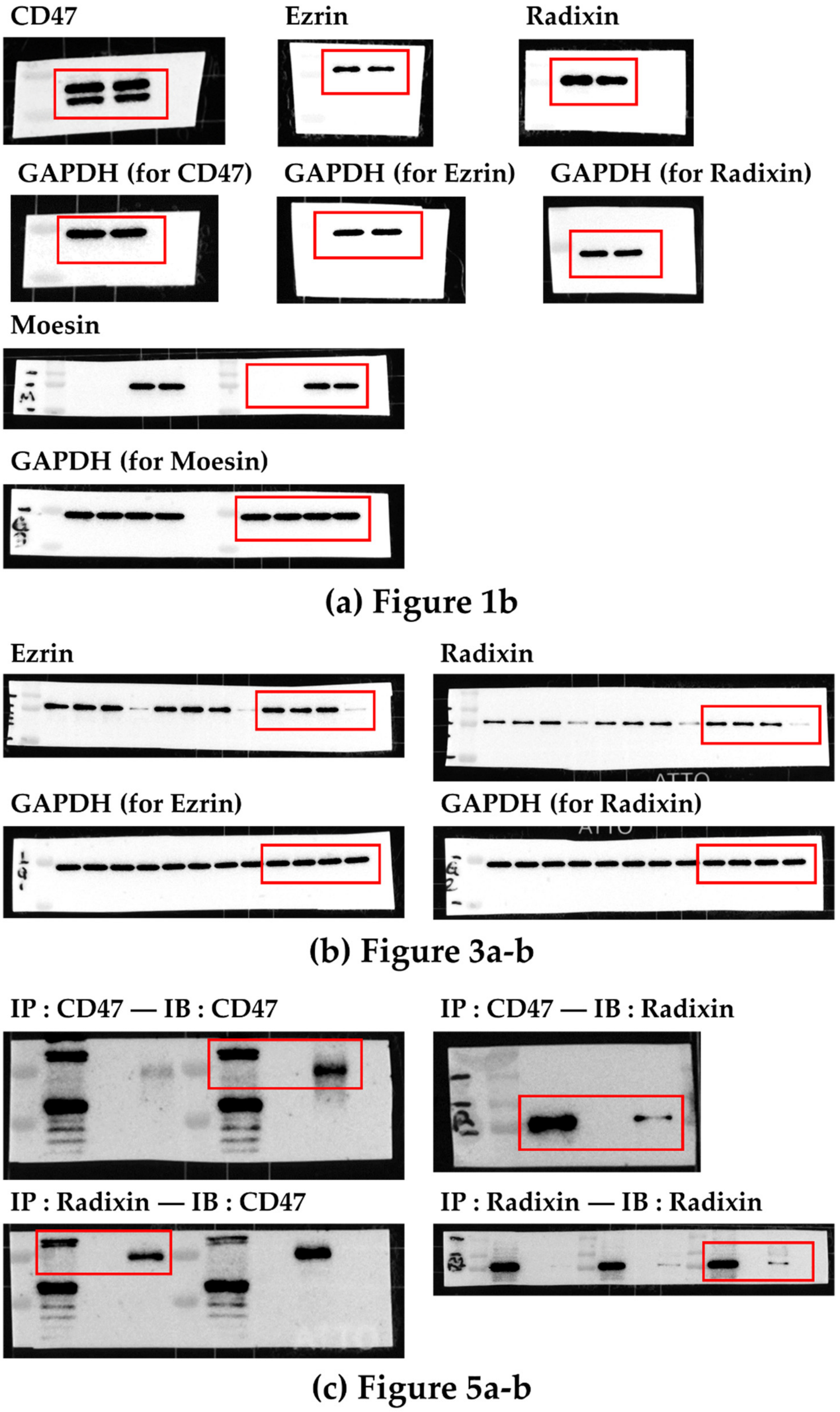

Figure S1. Original immunoblot images. The original western blotting membrane for CD47, ezrin, radixin, and moesin as well as the corresponding glyceraldehyde-3-phosphate dehydrogenase (GAPDH) as a loading control shown in (**a**) Figure 1b, (**b**) Figure 3a-b, and (**c**) Figure 5a-b. Each image in the red box corresponds to that in the main manuscript.

Negative fluorescence staining of no-primary-antibody control KP-2 cells

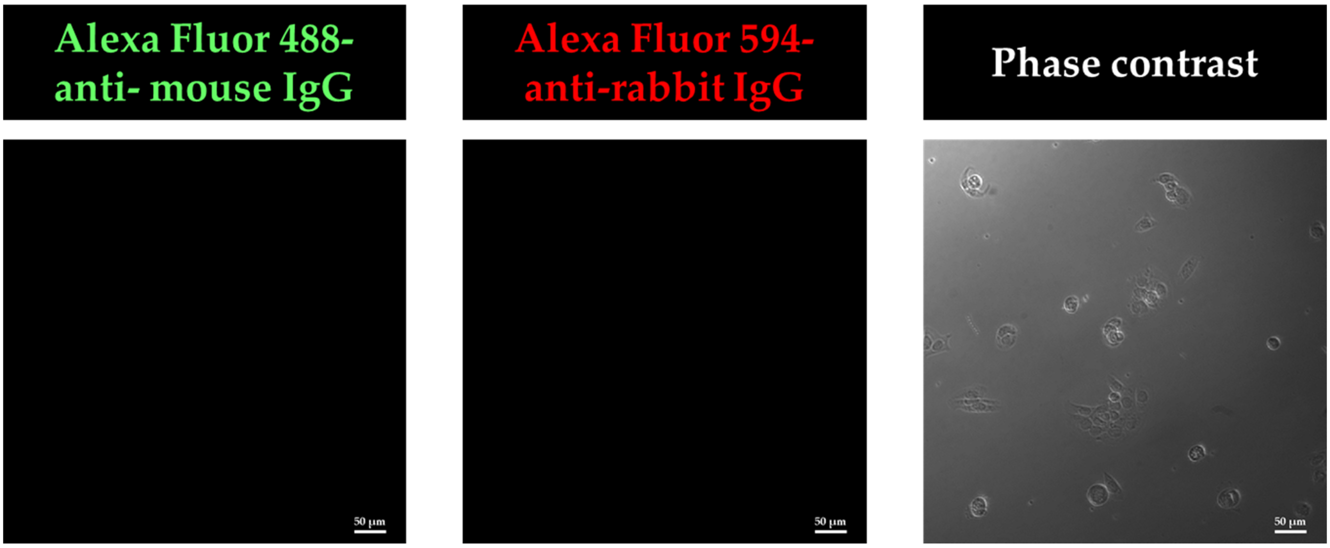

Figure S2. Negative fluorescence staining of no-primary-antibody control KP-2 cells. Left; Fluorescence image of goat anti-mouse IgG (H+L) secondary antibody conjugated with an Alexa Fluor 488 without any primary antibodies, Middle; Fluorescence image of goat anti-rabbit IgG (H+L) secondary antibody conjugated with an Alexa Fluor 594 without any primary antibodies, Right; Phase-contrast image. Scale bars: 50 µm. All images were captured by confocal laser scanning microscopy.

Gene expressions of CD47, ezrin, radixin, and moesin (ERM) in KP-2 cells

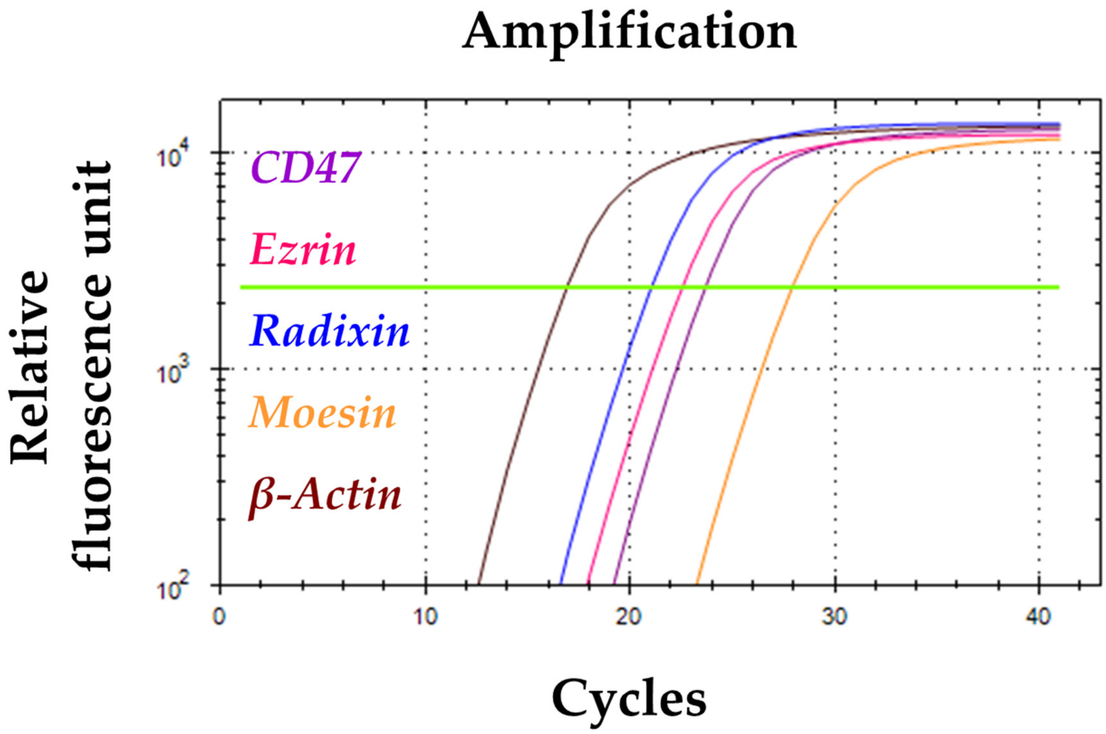

Figure S3. Gene expressions of CD47, ezrin, radixin, and moesin (ERM) in KP-2 cells. Representative amplification curve for mRNA expressions of CD47, ezrin, radixin, and moesin as well as β-actin in KP-2 cells as determined by real-time reverse transcription polymerase chain reaction (RT-PCR). Data are representative of three independent experiments using at least three independent samples of total RNA extracted from cells.

Counterstaining of CD47 with F-Actin in KP-2 cells

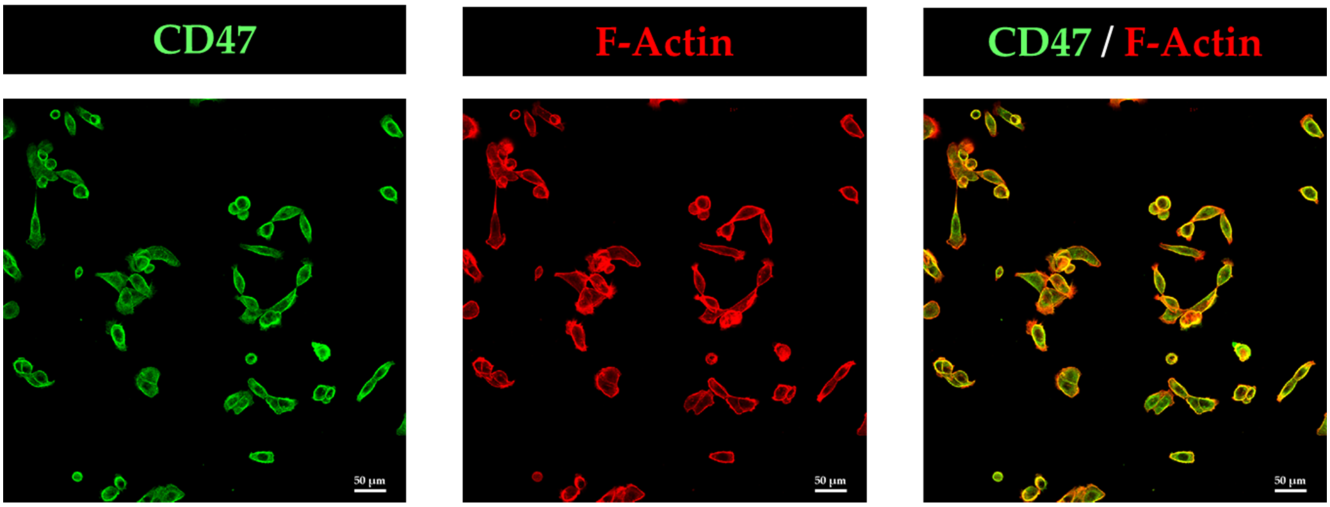

Figure S4. Counterstaining of CD47 with F-Actin in KP-2 cells. CD47 labeled with Alexa Fluor 488 (green) physically colocalized with F-actin labelled with phalloidin conjugated to the tetramethylrhodamine (TRITC) (red), which binds F-actin with high selectivity and affinity, implying the cellular membrane localization od CD47. Scale bars: 50 µm. All images are representative of at least three independent experiments.

Cell viability of KP-2 cells exposed to treatment with siRNAs

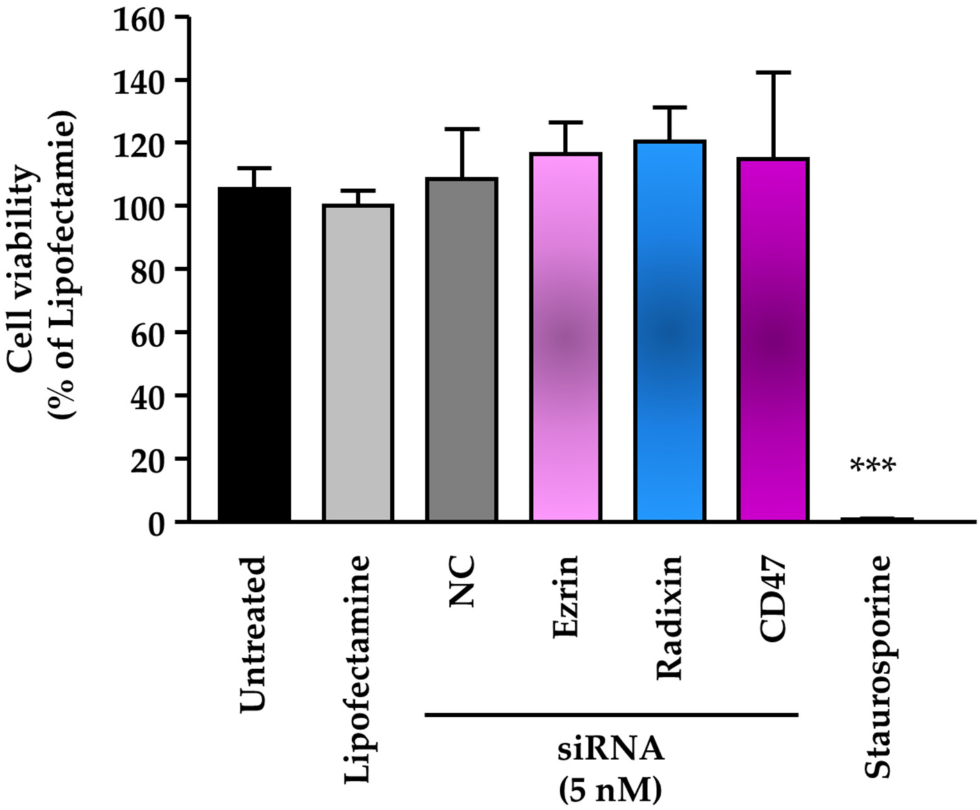

Figure S5. Cell viability of KP-2 cells exposed to treatment with siRNAs. Cells were cultured with transfection medium (Untreated), transfection reagent (Lipofectamine), non-targeting control (NC) siRNA, or siRNAs against each target gene at 5 nM for three days. Cell viability was assessed with the PrestoBlue cell viability reagent. Staurosporine at the final concentration of 1.0 µM is included as a positive control to strongly reduce *in vitro* cell viability. $n = 6$, ***$p < 0.001$ vs. Lipofectamine. All data were expressed as the mean ± SEM and analyzed by one-way ANOVA followed by Dunnett's test.

Reverse immunoprecipitation with an anti-radixin antibody to detect the molecular interaction between radixin and CD47 in KP-2 cells

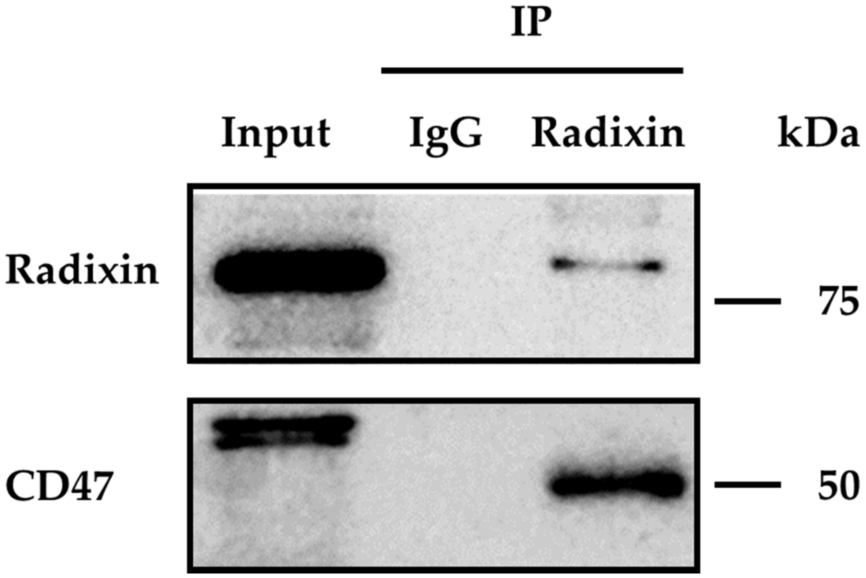

Figure S6. Reverse immunoprecipitation with an anti-radixin antibody to detect the molecular interaction between radixin and CD47 in KP-2 cells. Representative immunoblots of CD47 and radixin in whole-cell lysates (input) and those co-immunoprecipitated with an anti-radixin antibody (Ab) or its isotype-matched control IgG Ab. IP: Immunoprecipitation, kDa: Molecular weights.

Materials and Methods for Supplementary Materials

1. Immunofluorescent Counterstaining of CD47 with F-Actin

KP-2 cells were seeded at a density of 1.0 × 10^5 cells on a polylysine-coated 35-mm glass bottom dish with an inner diameter of 14 mm (Matsunami Glass, Kishiwada, Japan) and incubated overnight at 37 °C under humidified conditions with 5% CO_2 to allow for attachment. The cells were washed with Dulbecco's phosphate saline (D-PBS) (FUJIFILM Wako Pure Chemical, Osaka, Japan) and fixed with 4% paraformaldehyde (FUJIFILM Wako Pure Chemical) at room temperature for 15 min, followed by washing thrice with D-PBS. Subsequently, the permeability of cellular membranes was increased with 0.5% Triton-X100 (Nacalai Tesque, Kyoto, Japan) at room temperature for 15 min. After that, to block non-specific protein–protein interactions, the cells were incubated in a blocking buffer containing D-PBS, supplemented with 10% normal goat serum (Thermo Fisher Scientific, Tokyo, Japan), 1% bovine serum albumin (BSA) (FUJIFILM Wako Pure Chemical), and 0.1% Tween-20 (FUJIFILM Wako Pure Chemical), at room temperature for 60 min. Thereafter, cells were incubated overnight at 4 °C under dark conditions with a mouse anti-CD47 antibody (Ab) (MA5-11895; Thermo Fisher Scientific, Tokyo, Japan) at a dilution of 1:50 in blocking buffer. After washing thrice with D-PBS supplemented with 0.1% Tween-20 (PBS-T), the cells were incubated for 60 min at room temperature with an Alexa Fluor 488-conjugated goat anti-mouse IgG (Heavy + Light chain) Ab (R37120; Thermo Fisher Scientific) at a dilution of 1 drop/500 μL in blocking buffer. After washing thrice with PBS-T, the plasma membranes were counterstained with an Actin Red 555 ReadyProbes Reagent (Thermo Fisher Scientific) at a dilution of 1 drop/1,000 μL in blocking buffer for 30 min at room temperature. The cells were washed thrice with PBS-T and then Fluoro-KEEPER Antifade Regent Non-Hardening Type (Nacalai Tesque) was added for storage and prevention of quenching. The preserved cells were observed and photographed at 0.5–1.0 μm intervals on the *z*-axis at an original magnification of × 20 using a Nikon Al confocal laser microscope system (Nikon Instrument, Tokyo, Japan). The three-dimensional images were reconstructed using the NIS-Elements Ar Analysis software (Nikon Instruments).

2. Cell Viability Assay

KP-2 cells were seeded at a density of 4.0 × 10^3 cells/well in 96-well cell culture plates (Corning, Glendale, AZ, USA) and were incubated overnight at 37 °C in a humidified atmosphere with 5% CO_2 to allow for attachment. Then, cells were treated with siRNAs at 5 nM as described in the Main Manuscript and 1.0 μM of staurosporine (Merck, Darmstadt, Germany), a suitable positive control to strongly reduce *in vitro* cell viability, for three days without exchanging medium. Subsequently, cells were incubated with a PrestoBlue Cell Viability Reagent (Thermo Fisher Scientific), a fast and sensitive assay for assessing cell viability [1,2], at 37 °C for 10 min under humidified conditions with 5% CO_2, protected from direct light. Thereafter, fluorescence signals were detected at wavelengths of 560 nm (excitation) and 590 nm (emission) using a Synergy HTX Multi-Mode Microplate Reader (Bio Tek Instrument, Winooski, VT, USA).

Table 1. Gene-specific primer sequences

Gene	Primer sequence (5′→3′)
h-β-Actin (forward)	TGGCACCCAGCACAATGAA
h-β-Actin (reverse)	CTAAGTCATAGTCCGCCTAGAAGCA
h-Ezrin (forward)	ACCATGGATGCAGAGCTGGAG
h-Ezrin (reverse)	CATAGTGGAGGCCAAAGTACCACA
h-Radixin (forward)	GAATTTGCCATTCAGCCCAATA
h-Radixin (reverse)	GCCATGTAGAATAACCTTTGCTGTC
h-Moesin (forward)	CCGAATCCAAGCCGTGTGTA
h-Moesin (reverse)	GGCAAACTCCAGCTCTGCATC
h-CD47 (forward)	GTTGAGAATTAGATCCCACATCGTA
h-CD47 (reverse)	AACCTTTAACGGTAACACAGCTGTA

Table 2. Source and dilution of antibodies

Antibodies	Source	Cat. No.	Dilution
Rabbit anti-ezrin	Cell Signaling Technology	3145	1:2,000 (WB)<br>1:50 (IF)
Rabbit anti-radixin	Gene Tex	GTX105408	1:2,000 (WB)<br>1:100 (IF)<br>5.0 μg/test (IP)
Rabbit anti-moesin	Cell Signaling Technology	3150	1:2,000 (WB)<br>1:50 (IF)
Mouse anti-CD47	Thermo Fisher Scientific	MA5-11895	1:300 (WB)<br>1:50 (IF)<br>4.0 μg/test (IP)
Rabbit (DA1E) mAb IgG XP Isotype Control	Cell Signaling Technology	3900	5.0 μg/test (IP)
Mouse (G3A1) mAb IgG1 Isotype Control	Cell Signaling Technology	5415	4.0 μg/test (IP)
Mouse anti- glyceraldehyde-3-phosphate dehydrogenase (GAPDH)	Merck	MAB374	1:20,000 (WB)
Alexa Fluor 488-conjugated goat anti-mouse IgG (heavy + light chain) ReadyProbes	Thermo Fisher Scientific	R37116	1 drop / 500 μL (IF)
Alexa Fluor 594-conjugated goat anti-rabbit IgG (heavy + light chain) ReadyProbes	Thermo Fisher Scientific	R37120	1 drop / 500 μL (IF)
HRP-conjugated anti-rabbit IgG (heavy + light chain)	SeraCare Life Sciences	5220-0336	1:10,000 (WB-ERM)
HRP-conjugated anti-mouse IgG (heavy + light chain)	SeraCare Life Sciences	5220-0341	1:10,000 (WB-GAPDH)
HRP-conjugated anti-mouse IgG (light chain specific) (D3V2A)	Cell Signaling Technology	58802	1:3,000 (WB-CD47)
APC-conjugated mouse anti-human CD47	BioLegend	323124	0.8 μg/test (FC)

western blotting; WB, immunofluorescence; IF, immunoprecipitation; IP, horseradish peroxidase; HRP, allophycocyanin; APC, flow cytometry; FC

References for Supplementary Materials

1. Lall, N.; Henley-Smith, C. J.; De Canha, M. N.; Oosthuizen, C. B.; Berrington, D., Viability Reagent, PrestoBlue, in Comparison with Other Available Reagents, Utilized in Cytotoxicity and Antimicrobial Assays. *Int. J. Microbiol.* **2013,** *2013*, 420601.
2. Boncler, M.; Rozalski, M.; Krajewska, U.; Podsedek, A.; Watala, C., Comparison of PrestoBlue and MTT assays of cellular viability in the assessment of anti-proliferative effects of plant extracts on human endothelial cells. *J. Pharmacol. Toxicol. Methods* **2014,** *69*, 9-16.
